# Supplementary material for: Dry Powder Comprised of Isoniazid-Loaded Nanoparticles of Hyaluronic Acid in Conjugation with Mannose-Anchored Chitosan for Macrophage-Targeted Pulmonary Administration in Tuberculosis
Source: Pharmaceutics. 2022 Jul 25;14(8):1543. doi: 10.3390/pharmaceutics14081543 (PMC9330414; doi:10.3390/pharmaceutics14081543)
Supplement: Supplementary file 1 [file pharmaceutics-14-01543-s001.zip › pharmaceutics-1796597-supplementary.pdf]

Supplementary file

Dry powder comprised of isoniazid loaded nanoparticles of hyaluronic acid in conjugation with mannose anchored chitosan for macrophage targeted pulmonary administration in Tuberculosis

Mahwash Mukhtar [1], Noemi Csaba [2,3], Sandra Robla [2,3], Rubén Varela-Calviño [4], Attila Nagy [5], Katalin Burian [6], Dávid Kókai [6], Rita Ambrus [1*]

[1]Institute of Pharmaceutical Technology and Regulatory Affairs, Faculty of Pharmacy, University of Szeged, Szeged, Hungary; mahwash.mukhtar@szte.hu

[2] Department of Pharmacology, Pharmacy and Pharmaceutical Technology, University of Santiago de Compostela, Santiago de Compostela, A Coruña, Spain; noemi.csaba@usc.es (N.C.); sandra.robla@outlook.es (S.R.)

[3] Center for Research in Molecular Medicine and Chronic Diseases, University of Santiago de Compostela, Santiago de Compostela, A Coruña, Spain; noemi.csaba@usc.es (N.C.); sandra.robla@outlook.es (S.R.)

[4] Department of Biochemistry & Molecular Biology, School of Pharmacy University of Santiago de Compostela, Santiago de Compostela, A Coruña, Spain; ruben.varela@usc.es

[5] Wigner Research Centre for Physics, Hungarian Academy of Sciences, Budapest, Hungary; nagy.attila@wigner.hu

[6] Department of Medical Microbiology, Albert Szent-Györgyi Medical School, University of Szeged, Szeged, Hungary; burian.katalin@med.u-szeged.hu (K.B.); kokai.david@med.u-szeged.hu (D.K.)

*Correspondence: ambrus.rita@szte.hu

Synthesis of mannosylated chitosan polymer (MC)

In our previous work, Thiolated chitosan (TC) was synthesized and later TC was conjugated with mannose to synthesize MC. Chitosan was dissolved in acetic acid to prepare 1% solution. Later, thioglycolic acid (TGA) was added to it along 50 mM (1-ethyl-3-3(3-dimethylamino propyl) carbodiimide hydrochloride)), EDAC to facilitate amide bond formation between TGA and chitosan by activation of carboxylic groups of TGA. This resulting suspension was dialyzed to remove free TGA. After three days, the product was freeze-dried to obtain white amorphous polymer, TC. Then, for the purpose of mannosylation, TC was solubilized to obtain 2% solution followed by the addition of 0.12 M cyanoborohydride to initiate reductive amination. 0.33 M mannose was added to the polymeric suspension and the final viscous mass was dialyzed by altering the media. It was dialyzed against methanol thrice, later using diethyl ether and finally with distilled water. Obtained product was freeze-dried and stored in light resistant and air-tight container.

Working of Aerodynamic profile testing system

The drug products were loaded into capsules and a Breezhaler dry powder inhalator device was used for the tests in the measurement setup shown in Figure S1. The measurement setup consists of a breath simulator, an induction port representing the upper respiratory tract, a vacuum pump with a critical flow controller and an Aerodynamic Particle Sizer (APS-TSI 3321 (TSI Incorporated, USA)). A constant air flow Q_2 was established in the system along the blue arrows using the pump (HCP5 High-capacity pump; Copley Scientific Ltd., UK) and flow controller (TPK 2000; Copley Scientific Ltd., UK). The compressor compensates for the Q_4 airflow taken by the particle counter and any losses. The airflow Q_5 provided by the compressor was determined by measuring the airflow through the upper inlet of the mixing unit, in the inhaler side, in the default condition. During the measurements, the breathing simulator produced the flow profile Q_3 activating the DPI unit through the mixing inlet (red arrows). The mixing inlet (Copley

Scientific Ltd., UK) provides an interface between the flow that activates the DPI and the main stream that transfers the particles to the APS. The APS samples particles from the main stream by an isokinetic nozzle.

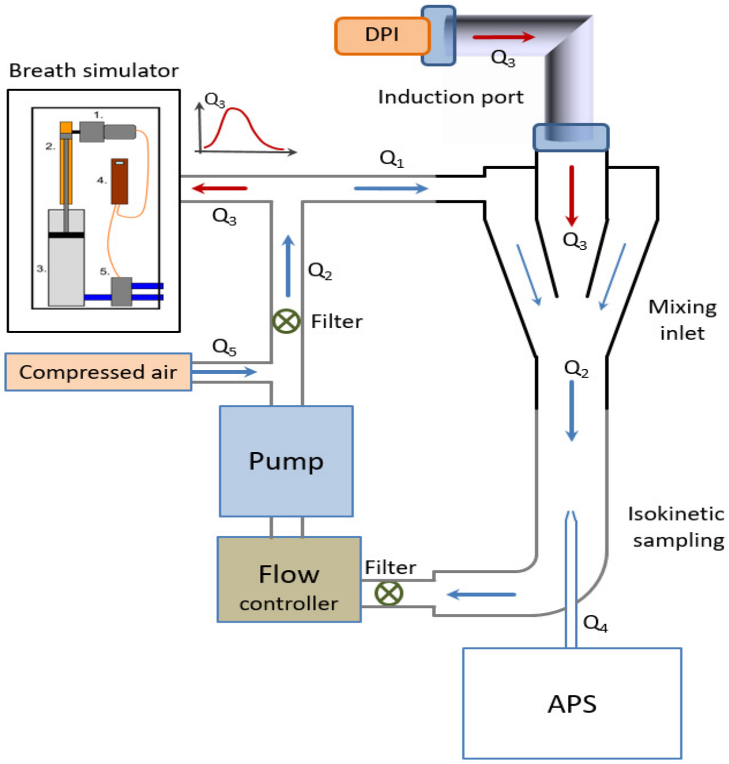

Figure S1: The schematic design and components of the measurement setup: DPI, induction port, APS, vacuum pump with a critical flow controller, mixing inlet, and PWG. The pulmonary waveform generator consists of: 1-Servo motor; 2-Timing belt; 3-Piston pump; 4-PLC; 5-Valves.

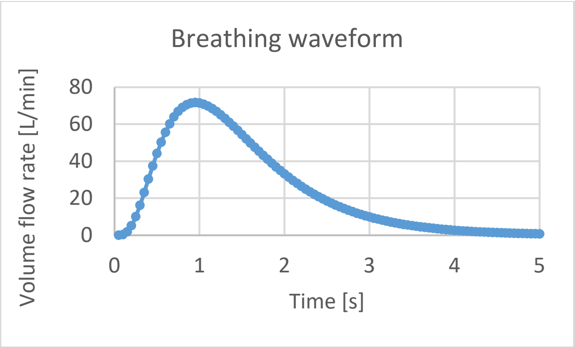

Figure S2: The applied breathing waveform with 50 ms time resolution

FTIR spectra

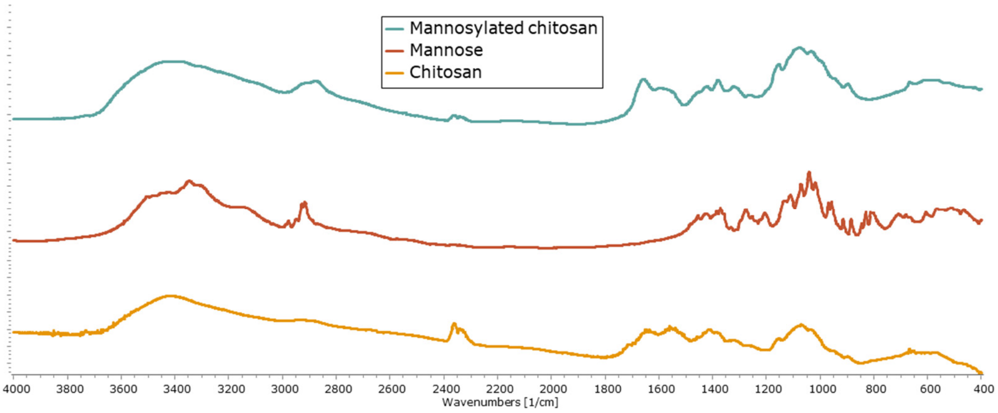

Figure S3: IR spectra of chitosan, mannose and mannosylated chitosan

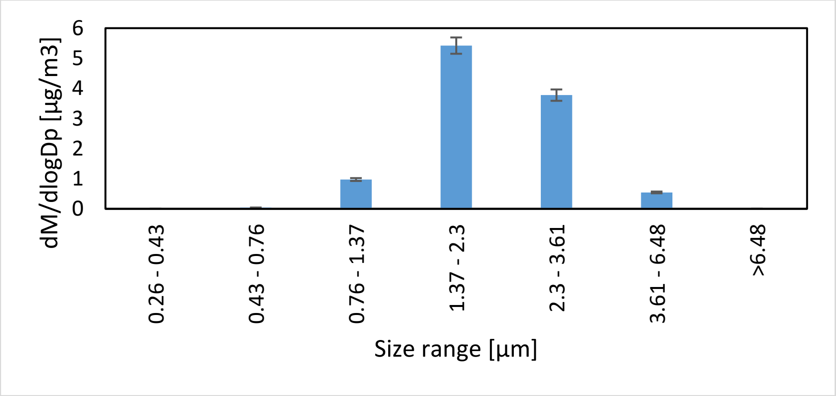

Figure S4: Calculated mass size distribution from the Aerodynamic Particle Sizer data by setting channel bounds according to the NGI cut-off sizes

Table S1: The results for the aerodynamic particle sizer for the nano dry powder INH-MC/HA, demonstrating the mass size distribution of particles, Results are expressed as mean ± S.D, n=4

	**Number Particle size**	**Surface Particle size**	**Mass Particle size**
**Median (µm)**	1.36 ± 0.028	1.89 ± 0.024	2.29 ± 0.013
**Mean (µm)**	1.47 ± 0.029	2.14 ± 0.047	2.88 ± 0.010
**Geometric mean (µm)**	1.35 ± 0.013	1.92 ± 0.011	2.42 ± 0.059
**Mode (µm)**	1.36 ± 0.001	1.81 ± 0.008	2.38 ± 0.018

**Geometric standard deviation**	1.50	1.56	1.71

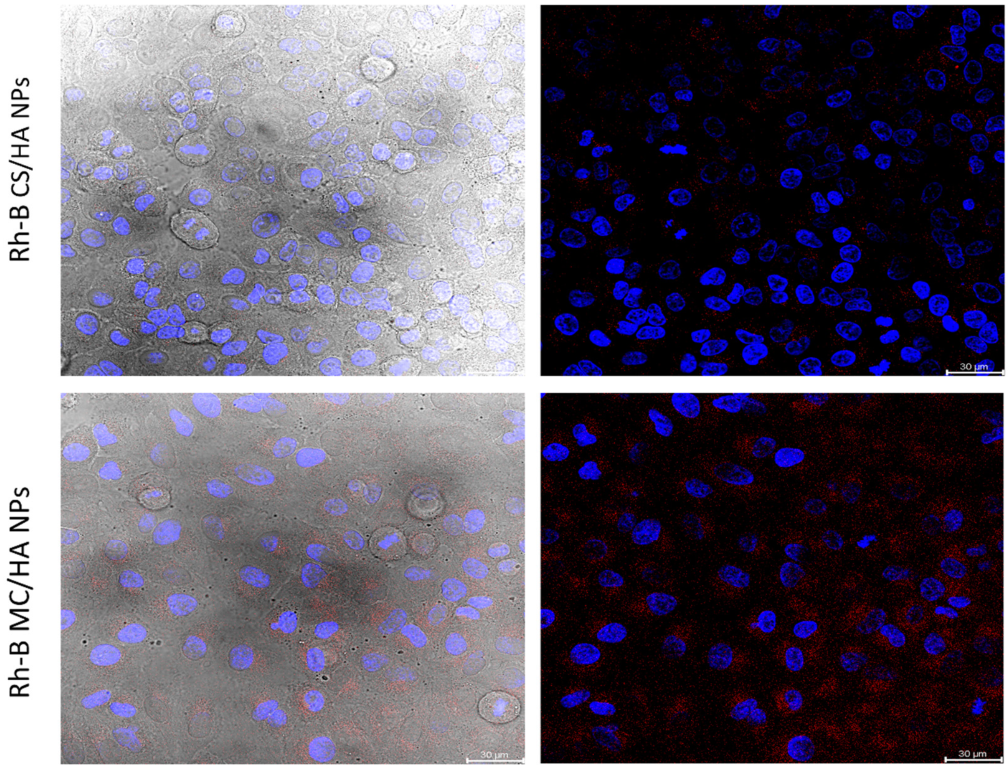

Figure S5: Merged bright-field (first row) and fluorescent confocal images (second row) acquired for Rh-B CS/HA NPs and Rh-B MC/HA NPs after incubation with the A549 cell line.

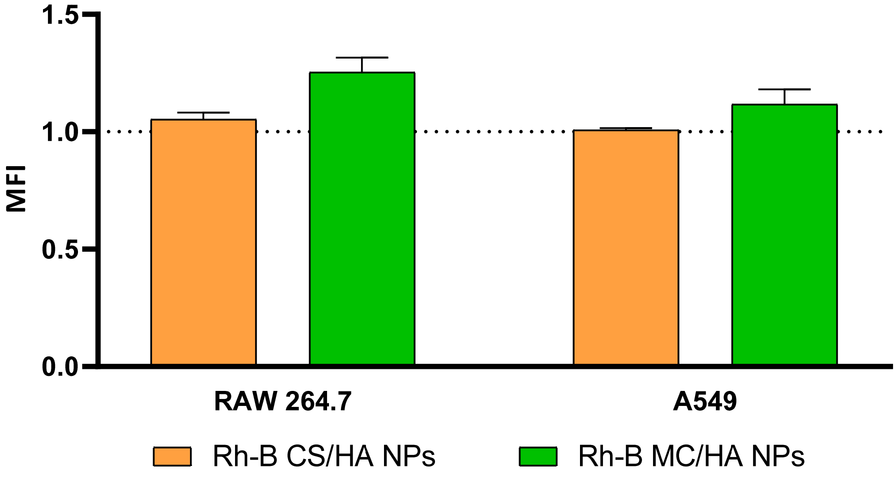

Figure S6: Quantification of uptake of NPs over 24 h by Flow cytometry (Detector FL3, Laser 488 nm, Filtre BP 575/25, Software: BD Spectra viewer, Rhodamine B 546/568 (Ex/Em)

Our flow cytometer is not the optimal one for Rhodamine B detection, because filters we use detect a field that does not fully encompass Rhodamine B.

Macrophage phenotype analysis

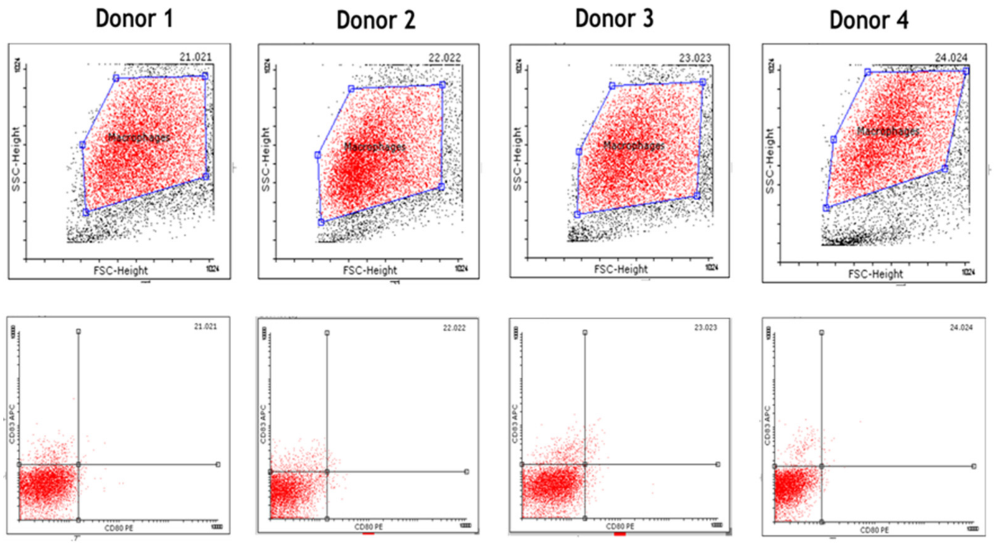

CONTROL

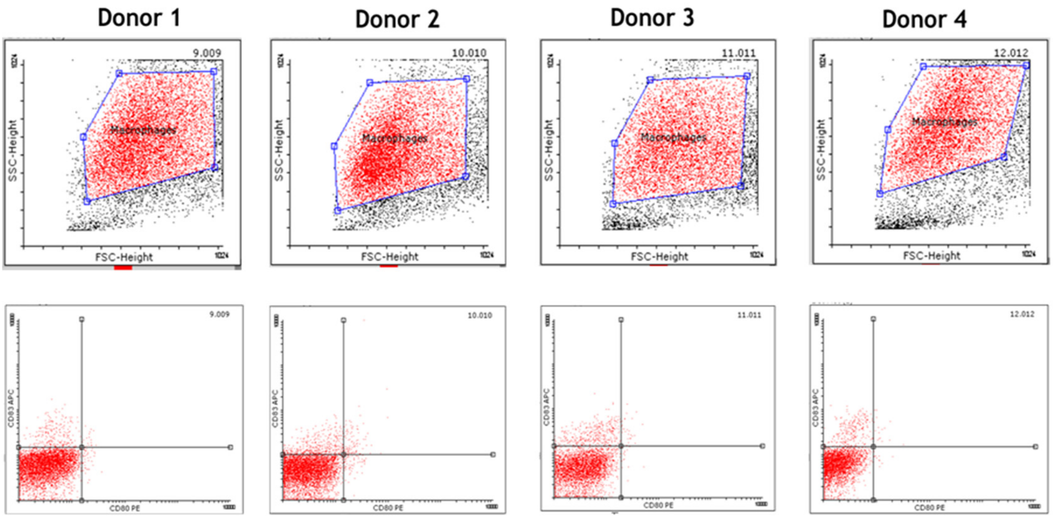

CS/HA NPs

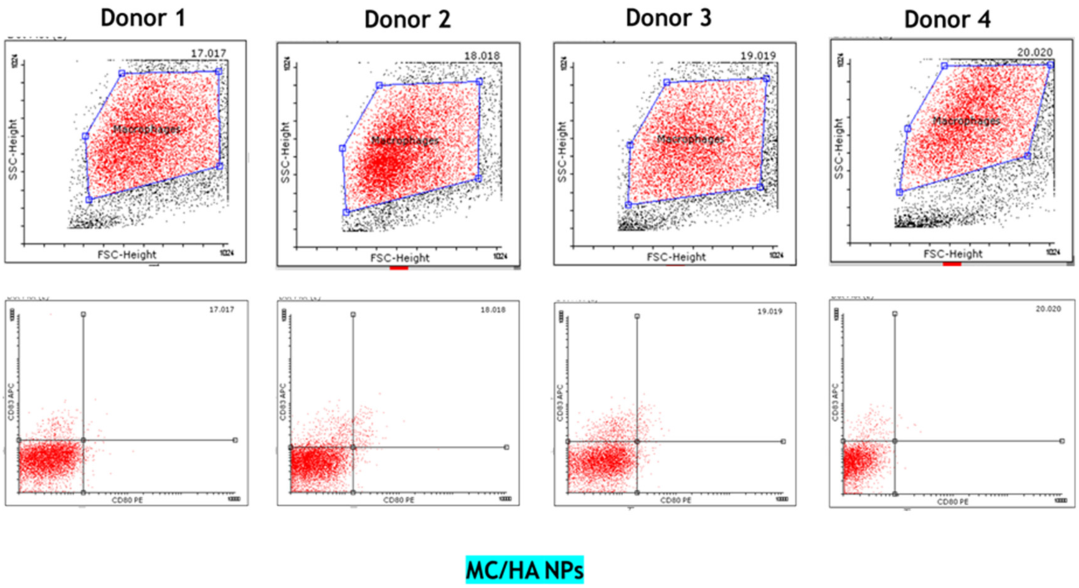

Figure S7: Human macrophage phenotype analysis- Expression of CD80 and CD83 (Flow cytometry data)
